# Expression of *Ascaris lumbricoides* putative virulence-associated genes when infecting a human host

**DOI:** 10.1186/s13071-021-04680-y

**Published:** 2021-03-23

**Authors:** Norashikin Mohd-Shaharuddin, Yvonne Ai Lian Lim, Romano Ngui, Sheila Nathan

**Affiliations:** 1 Department of Parasitology, Faculty of Medicine, University of Malaya, Kuala Lumpur, Malaysia; 2 Department of Biological Sciences and Biotechnology, Faculty of Science and Technology, Universiti Kebangsaan Malaysia, Selangor, Malaysia

**Keywords:** *Ascaris lumbricoides*, Virulence-associated, Immune response, Helminths

## Abstract

**Background:**

*Ascaris lumbricoides* is the most common causative agent of soil-transmitted helminth infections worldwide, with an estimated 450 million people infected with this nematode globally. It is suggested that helminths are capable of evading and manipulating the host immune system through the release of a spectrum of worm proteins which underpins their long-term survival in the host. We hypothesise that the worm overexpresses these proteins when infecting adults compared to children to cirvumvent the more robust defence mechanisms of adults. However, little is known about the parasite’s genes and encoded proteins involved during *A. lumbricoides* infection. Hence, this study was conducted to assess the expression profile of putative virulence-associated genes during an active infection of adults and children.

**Methods:**

In this study, quantitative PCR was performed to evaluate the expression profile of putative virulence-associated genes in *A. lumbricoides* isolated from infected children and adults. The study was initiated by collecting adult worms expelled from adults and children following anthelminthic treatment. High-quality RNA was successfully extracted from each of six adult worms expelled by three adults and three children, respectively. Eleven putative homologues of helminth virulence-associated genes reported in previous studies were selected, primers were designed and specific amplicons of *A. lumbricoides* genes were noted. The expression profiles of these putative virulence-associated genes in *A. lumbricoides* from infected adults were compared to those in *A. lumbricoides* from infected children.

**Results:**

The putative virulence-associated genes *VENOM*, *CADHERIN* and *PEBP* were significantly upregulated at 166-fold, 13-fold and fivefold, respectively, in adults compared to children. Conversely, the transcription of *ABA-1* (fourfold), *CATH-L* (threefold) and *INTEGRIN* (twofold) was significantly suppressed in *A. lumbricoides* from infected adults.

**Conclusions:**

On the basis of the expression profile of the putative virulence-associated genes, we propose that the encoded proteins have potential roles in evasion mechanisms, which could guide the development of therapeutic interventions.

**Graphical Abstract:**

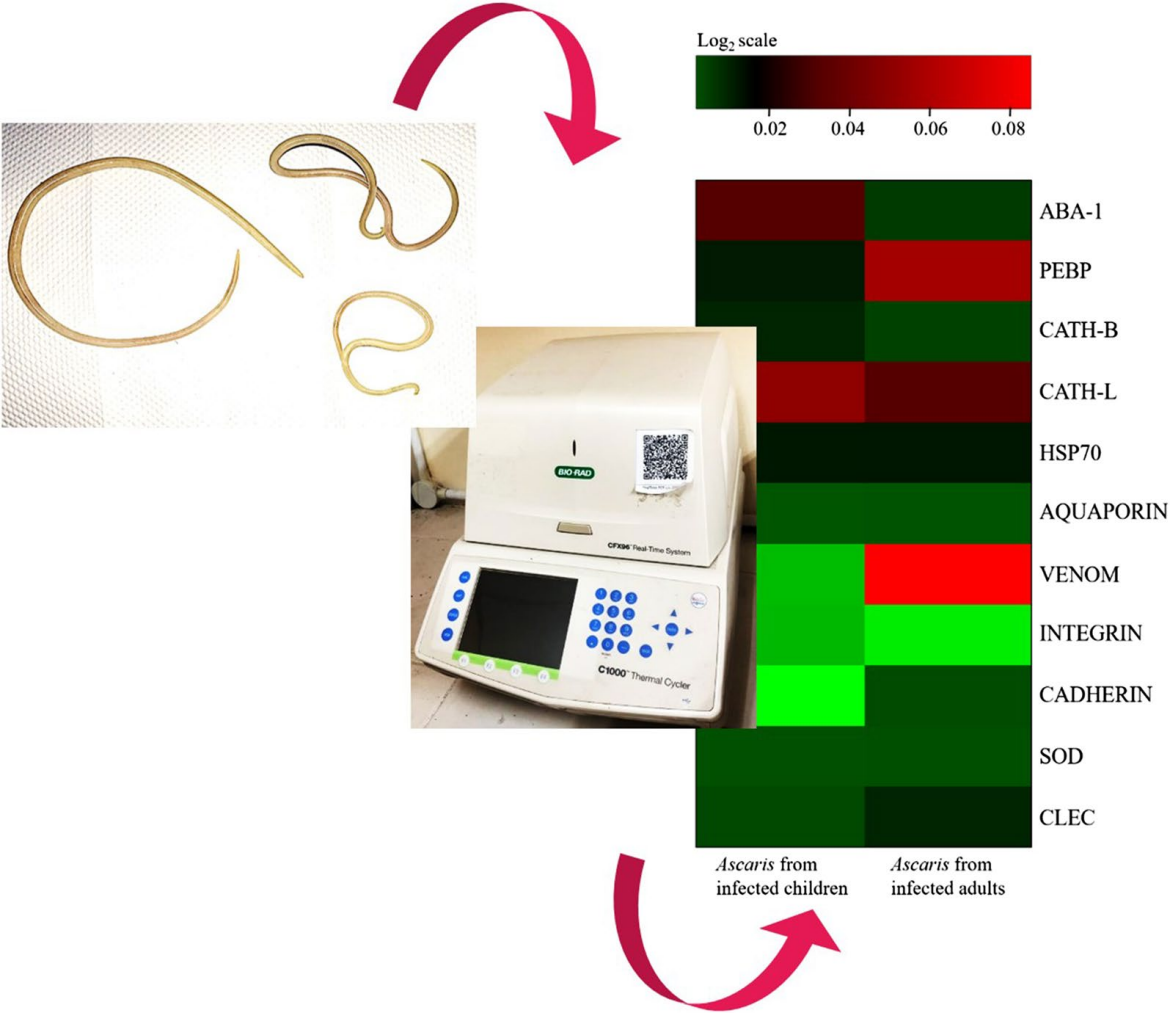

**Supplementary Information:**

The online version contains supplementary material available at 10.1186/s13071-021-04680-y.

## Background

*Ascaris lumbricoides* is a major causative agent of soil-transmitted helminth (STH) infections worldwide. According to the Global Burden of Diseases Study, in 2019, a total of 446 million cases of *A. lumbricoides* infection were reported globally, involving both males and females [1]. In Malaysia, the incidence of *A. lumbricoides* infection is significant and highly prevalent among the indigenous (*Orang Asli*) communities [2–4]. Typically, *A. lumbricoides* infection is asymptomatic. However, in heavily infected individuals, the infection can lead to upper gastrointestinal bleeding, small bowel obstruction, volvulus, intussusception, peritonitis and gastric ascariasis [5–7]. Chemotherapy (albendazole, mebendazole, levamisole and pyrantel pamoate) is routinely prescribed to curb ascariasis, particularly among children. Nonetheless, re-infection by STHs in endemic areas where clean water, sanitation and hygiene is lacking is a major drawback to controlling STHs, especially *A. lumbricoides*. A previous local study among the *Orang Asli* school children of Peninsular Malaysia reported that re-infection with *A. lumbricoides* at 6 months post-treatment was close to the baseline situation [8]. Meanwhile, in eastern Indonesia, a study described that the prevalence of *A. lumbricoides* was significantly higher than the baseline prevalence 34 months after the cessation of the mass drug administration programme [9], while in the People’s Republic of China, re-infection with *A. lumbricoides* was reported as early as 4 months post-drug administration [10].

Environmental and behavioural features as well as host susceptibility and genetics play an important role in the chronicity and survival of parasites. Parasitic helminths’ capacity to manipulate and modulate host immunity underpins their long-term survival in the host [11, 12], and also influences the capability of the host to mount effective responses towards the parasite. Understanding parasite evasion strategies in the host during infection is a compelling area of research in terms of novel drug and vaccine development. It has been proposed that the parasite evasion strategies are orchestrated through the release of molecules during the host–parasite interaction. Significant attention has focussed on the ‘excretory/secretory’ (ES) antigens of helminths. These ES products contribute to immune evasion strategies of the parasites through mechanisms such as shedding of surface-bound ligands and cells, alterations of lymphocyte, macrophage and granulocyte functions and modulation of other host inflammatory responses [13, 14].

With the advancement in helminth genomics, studies on ES products have revealed a set of proteins secreted by helminths, including proteases, protease inhibitors, venom-allergen homologues, glycolytic enzymes and lectins [15]. The composition of these proteins has been characterised in *Brugia malayi* [16], *Necator americanus* [17], *Toxocara canis* [18], *Ancylostoma caninum* [19] and *Fasciola hepatica* [20]. However, knowledge of the possible virulence mechanisms involved during *A. lumbricoides* infection is limited. *Ascaris suum* is a closely related species of *A. lumbricoides*, and in this study we proposed that homologues of *A. suum* virulence genes [21] exist in *A. lumbricoides*; these genes are henceforth referred to as putative *A. lumbricoides* virulence-associated genes. We examined the expression of 11 potential *A. lumbricoides* virulence-associated genes during an infection of adults and children.

Human protective immunity against helminths is well developed through a spectrum of immune responses, especially after prolonged exposure to the parasites. The T-helper 2 cell (Th2)-mediated immune response plays an important role in protection against intestinal helminth infection through the activation of immune-related cells [22]. While healthy adults have an established immune defence system, young children are more vulnerable to infection [23]. Nonetheless, a strong relationship between humoral immune responses and current or future worm burdens has yet to be established [24]. This study was conducted to compare the expression levels of *A. lumbricoides* putative virulence-associated genes from infected children and adults. We hypothesised that the expression of virulence-associated genes will be detectable in the worms obtained from both adults and children; nevertheless, the expression of these genes may be significantly higher in adults compared to children. Virulence-associated genes that are differentially modulated in the host or their encoded proteins could be key targets for the development of new drugs and vaccines. Thus, in this study we compared the expression levels of *A. lumbricoides* putative virulence-associated genes collected from infected Malaysian indigenous adults and children.

## Methods

### Gene annotation of *A. lumbricoides* putative virulence-associated genes and primer design

Given the paucity of information for virulence-associated genes in *A. lumbricoides* when this project was initiated, information related to this study was inferred from previous studies on *Toxocara* spp. [25, 26]. Comparison of *Toxocara canis* draft genome sequences with those of other nematodes showed that *T. canis* genes have the highest sequence similarity to *Ascaris suum*, with 67.5% of the predicted *T. canis* genes having an orthologue in *A. suum* (*n* = 11,658; 62.7%) [26]. The *A. suum* genome has been sequenced [21], and this parasite is routinely used as a research model for its close relative, *A. lumbricoides*. *Toxocara canis* protein-encoding sequences were obtained from the UniProt database and subjected to a BLASTX search to identify similar protein sequences from either *A. lumbricoides* or *A. suum* (based on the availability). The *A. lumbricoides* putative virulence gene homologues were retrieved from the National Center for Biotechnology Information (NCBI) with the majority of genes uncharacterised (Table 1). A protein domain search was performed using the Conserved Domain Database (CDD) [27]. To better understand the functional distribution, Gene Ontology (GO) analysis was performed to annotate all the putative genes into three high level categories: Biological Process, Molecular Function and Cellular Component. For this analysis, the GO term ‘option’ was used to predict functions with default parameters and GO terms were plotted using Blast2Go [28].

**Table 1:** Putative *Ascaris lumbricoides* virulence-associated genes

Genes	Abbreviations^a^	Accession no. (as of August 2016)
ABA-1 allergen	*ABA-1*	U86097
Phosphatidylethanolamine-binding proteins	*PEBP*	ERG86178
Cathepsin-B	*CATH-B*	U51892
Cathepsin-L	*CATH-L*	AY069923
Heat Shock Protein 70 kDa	*HSP70*	ERG79780
Aquaporin 3	*AQUAPORIN*	ERG85246
Venom allergen 3	*VENOM*	ERG80545
Integrin alpha	*INTEGRIN*	ERG80089
Cadherin	*CADHERIN*	ERG80754
Superoxide dismutase	*SOD*	ERG81337
C-type lectin	*CLEC*	ERG84673

^a^The abbreviations used are for this study only

### Collection of adult *A. lumbricoides* and experimental design for real-time PCR (quantitative PCR)

Prior to data collection, the study protocol was approved by the Ethics Committee of the University of Malaya Medical Centre (UMMC), Malaysia (MEC ID Number: 20144-104). Based on Kato-Katz data on indigenous people residing in Selangor, Peninsular Malaysia (Additional file 1: Table S1), a total of 14 participants with heavy and moderate infection were identified and prescribed a 3-day course of 400 mg/daily albendazole tablets (Zentel®; GlaxoSmithKline [GSK], London, UK). The participants were instructed to store the expelled worms in containers with holes, which were provided to them. As the worms were expelled from individual participants at different times, the adult worms were collected between 1 and 3 h post-expulsion. The worms were rinsed with distilled water and snap-frozen in liquid nitrogen. The containers with the worms were stored in liquid nitrogen dewars and transported to the laboratory where they were stored − 80 °C prior to RNA extraction.

### Isolation of total RNA and cDNA synthesis

Extraction of *A. lumbricoides* RNA was conducted using RNAse-free consumables and tools such as blades, pestle and mortar, forceps and spatula. The anterior part of the harvested frozen *A. lumbricoides* adults was crushed into powder form using a RNAse-free pestle and mortar and homogenised in 15 ml TRIzol (Invitrogen™, Thermo Fisher Scientific, Carlsbad, CA, USA). The RNA was extracted using TRIzol, treated with DNase (Qiagen, Hilden, Germany) to remove contaminating genomic DNA and purified using the RNeasy® Mini Kit (Qiagen) according to the manufacturer’s protocol. RNA integrity and concentration were determined using the Agilent 2100 Bioanalyzer (Agilent Technologies Inc., Santa Clara, CA, USA) and Nanodrop ND-1000 spectrophotometer (NanoDrop Technologies Inc., Wilmington, DE, USA), respectively. First-strand cDNA was synthesised from 1 µg of total RNA of each sample using Superscript IV transcriptase following the manufacturer’s instructions (Invitrogen). The synthesised cDNA samples were stored at − 20 °C until further use.

### Primer design and validation

The design of primers for the predicted *A. lumbricoides* virulence-associated genes was guided by the sequences of *T. canis* and *A. suum* homologues using Oligo Explorer version 1.1.2 (http://oligoexplorer.software.informer.com/) and synthesised by First BASE Laboratories (Selangor, Malaysia) followed by* in silico* PCR for verification of primer efficiency (Table 2). To obtain an optimum annealing temperature for the primers, PCR amplification was performed in a total volume of 20 µl reaction mixture containing 10 µl master mix (GeNet Bio, Chungcheongnam-do, South Korea), 2 µl of each forward and reverse primer (10 µM), 4 µl of distilled water and 2 µl of cDNA template. PCR cycling was carried out in the T100™ Thermal Cycler (Bio-Rad Laboratories, Inc., Hercules, CA, USA) under the following conditions were: initial denaturation at 94 °C, 5 min; then denaturation at 94 °C/30 s, annealing at a temperature gradient from 55 to 60 °C/30 s and extension at 72 °C/30 s, for 35 cycles; and a final extension at 72 °C for 7 min. After several trials, the optimum annealing temperature was achieved at 57 °C. The PCR products were visualised on a gel imager.

**Table 2:** Primers used in quantitative PCR

Genes	Sequences (5′–3′)	Product size (bp)	Tm (°C)
*β-actin*	F: 5′-CTCGAAACAAGAATACGATG-3′ R: 5′-ACATGTGCCGTTGTATGATG-3′	450	57
*18S*	F: 5′-ATCGGTCGCGTAGGGTGGCT-3′ R: 5′-AAGCCGCAGGCTCCACTCCT-3′	200	57
*ABA-1*	F: 5′-ACAACAAGCAACAGAAAAGC-3′ R: 5′-TGACCTCGGAAAGCATCT-3′	155	57
*PEBP*	F: 5′-CGATAGTGGCGTTGAGGT-3′ R: 5′-GGAATTTGGGGGTTTCAC-3′	152	57
*CATH-B*	F: 5′-GCTGCTGTAAAAGTTGTG-3′ R: 5′-AAGGTGGGAAAGGATAAG-3′	138	57
*CATH-L*	F: 5′-AGGCAAGGAGATGAAGTG-3′ R: 5′-GCATTGTGGCTCGTAGTA-3′	196	57
*HSP70*	F: 5′-TACAACAAAGGCAAACTCAC-3′ R: 5′-CTCGCATTCATCCAAAAG-3′	171	57
*AQUAPORIN*	F: 5′-GGAAGAGTGAGGCGAAAT-3′ R: 5′-CCGATGAACAGAAGCAAG-3′	130	57
*VENOM*	F: 5′-TCAGAGGTGGACGACTAT-3′ R: 5′-GACAAACGACAATGATACTG-3′	172	57
*INTEGRIN*	F: 5′-ATCAACACCCGAGCAACT-3′ R: 5′-AGCCAAGCACCACTAACTG-3′	144	57
*CADHERIN*	F: 5′-GTCAGGTCTCGTCAATCG-3′ R: 5′-TGGCACTTCAACATCGTAG-3′	183	57
*SOD*	F: 5′-CCCGATTTACCATACGAC-3′ R: 5′-CCACCACCATTGAACTTC-3′	197	57
*CLEC*	F: 5′-CGGCTTTGACGAGATAGAG-3′ R: 5′-AAGACCACCGACCAGTTT-3′	182	57

F, Forward; R, Reverse; Tm, primer melting temperature

### Real-time PCR (quantitative PCR) and data analysis

Eleven putative *A. lumbricoides* or *A. suum* gene sequences were selected based on their homology to *T. canis* virulence-associated genes reported in previous studies (Table 1). A tenfold serial dilution was performed to generate a standard curve by plotting the quantification cycle (C_q_) for each dilution point against the starting quantity of cDNA (100 to 0.1 ng) to validate the efficiency of each primer set. Standard curve analysis and quantitative PCR (qPCR) reactions were performed using Ssofast™ Evagreen® (Bio-Rad Laboratories) according to the manufacturer’s protocol on the CFX96 Touch™ Real-Time PCR Detection System (Bio-Rad Laboratories). Standard curves were computed automatically on the average normalised C_q_ value using the same real-time PCR system. The qPCR programme used was: an initial denaturation at 95 °C, 1 min; followed denaturation at 95 °C/30 s, annealing at 57 °C/30 s and extension at 76 °C/30 s, for 40 cycles; with a final extension at 60 °C for 7 min. Data from each of the assays were normalised to the housekeeping or reference genes β-actin [29] and 18S ribosomal RNA [30]. At the end of each cycle, melting curve analysis of the primers was performed by increasing the temperature by 0.5 °C from 55 °C to 95 °C to ensure only specific products were obtained with no formation of primer dimers. Each amplification reaction was performed in triplicate and no-template control (NTC) was included in each of the assays to monitor cross-contamination. The average C_q_ value for each treatment group was calculated from an analysis of experimental triplicates. The expression level of the genes of interest was calculated manually using the formula 2^−ΔΔCq^ [31]. A probability level of *P* < 0.05 was considered significant.

## Results

### Sample collection and RNA extraction

**Fig. 1:**
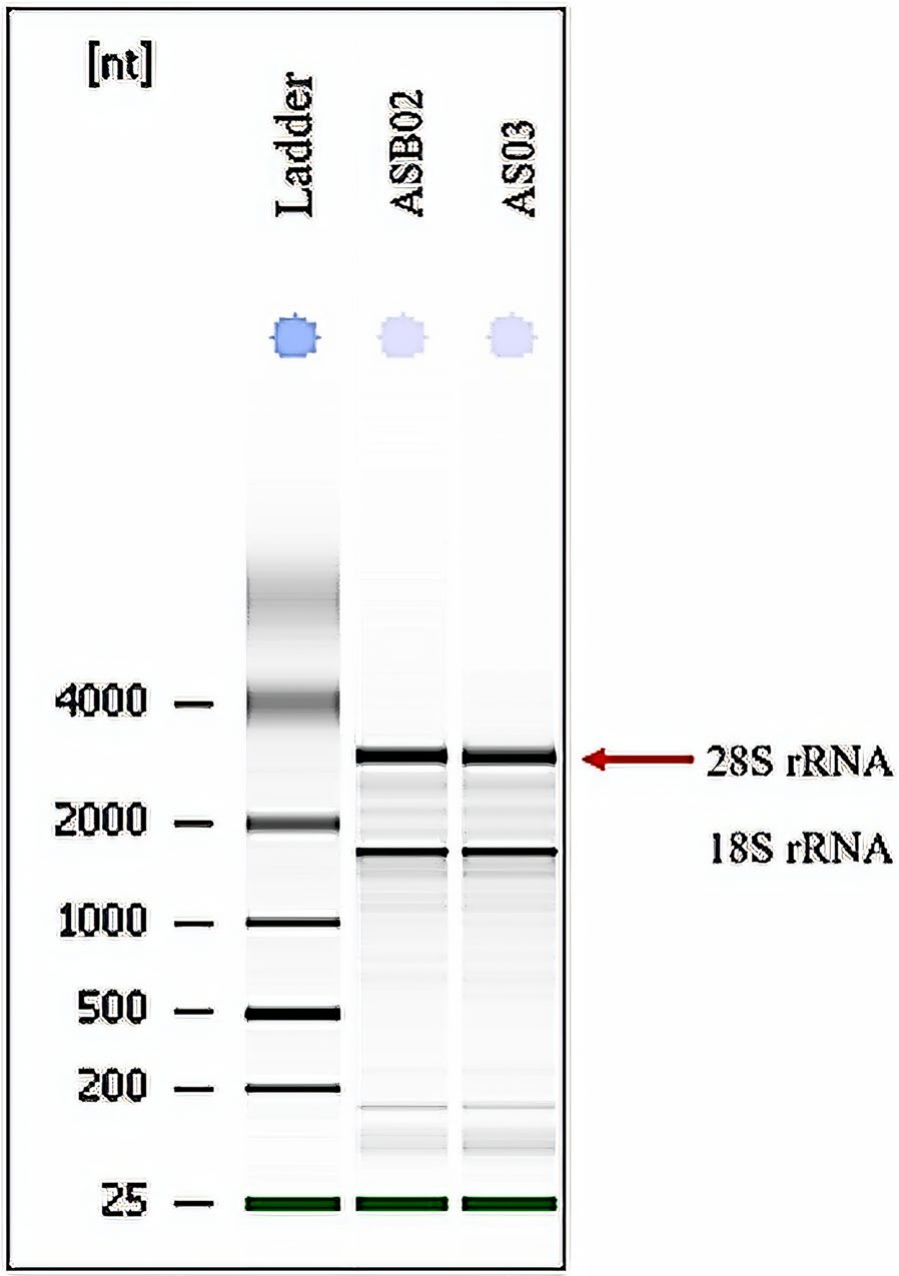
Representative electrophoresis image of total RNA extracted from two biological replicates of adult *Ascaris lumbricoides* (AsB02 and As03), showing two distinct bands representing 28S and 18S rRNA

Three to five days after the 3-day anthelminthic prescription, a total of 14 adult *A. lumbricoides* were obtained from two groups of participants: (i) children (aged between 2 and 5 years old) and (ii) adults (aged ≥ 15 years). Each individual expelled between one and two adult worms. The category of participants was made based on the availability of the samples due to the limited number of adult worms expelled from the hosts. We noted that two of the worms were female, and the rest were identified as male worms. All of the worms were of a similar size irrespective of sex and whether they were expelled by infected adult or infected child. Total RNA of individual adult *A. lumbricoides* expelled from infected children and adults was prepared and visualised on the Agilent 2100 Bioanalyzer. Based on this analysis, only six of the RNA samples (extracted from 3 worms collected from 3 children and 3 worms from 3 adults) were deemed suitable for gene expression analysis. Two distinct bands, representing 28S and 18S rRNA, were noted for these six selected samples (Fig. 1). The RNA integrity number (RIN) of the extracted *A. lumbricoides* total RNA ranged from 6.5 to 7.7.

### Modulation of *A. lumbricoides* putative virulence-associated genes expression

**Fig. 2:**
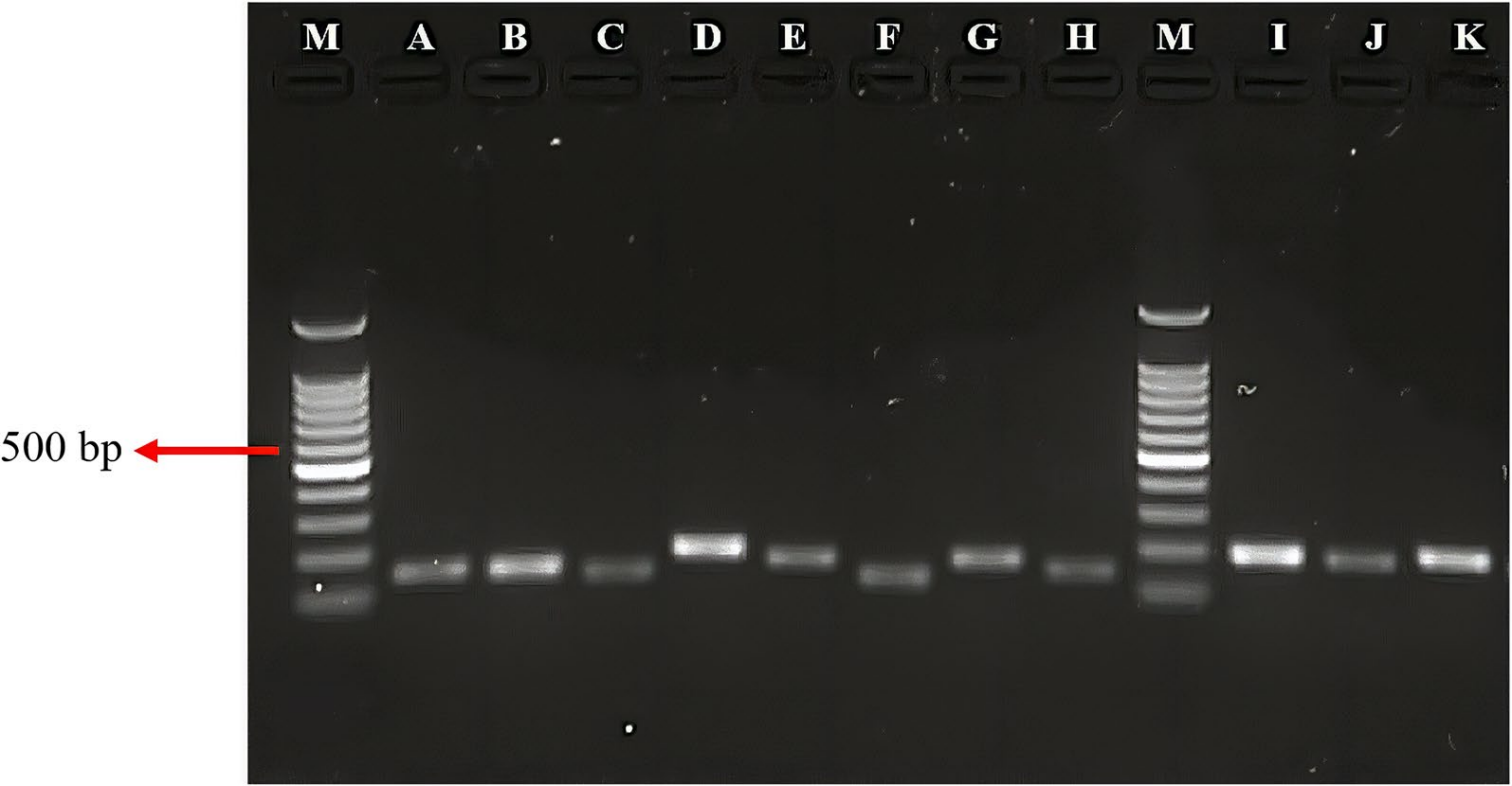
Specific amplicons of putative virulence-associated genes amplified from *A. lumbricoides* cDNA. Lanes:* M* 100-bp ladder,* A* *ABA-1* (155 bp),* B* *PEBP* (152 bp),* C* *CATH-B* (138 bp),* D* *CATH-L* (196 bp),* E* *HSP70* (171 bp),* F* *AQUAPORIN* (130 bp),* G* *VENOM* (172 bp),* H* *INTEGRIN* (144 bp),* I* *SOD* (197 bp),* J* *CADHERIN* (183 bp),* K* *CLEC *(182 bp)

Eleven putative virulence-associated genes were selected; these genes encoded proteins of various functions based on previous studies on *T. canis*. Of these 11 genes, only eight were successfully annotated with predicted GO terms (Table 3). The predicted functions of these genes are listed in Additional file 1: Table S2. When we initiated this study, the selected *A. lumbricoides* genes had not been previously annotated due to the absence of a complete genome sequence and are therefore referred to here as putative virulence-associated genes. In lieu of this limitation, the designed primers were subjected to an initial screen by gradient PCR. Specific amplicons with a common annealing temperature of 57 °C were clearly observed for all 11 genes (Fig. 2). *Ascaris lumbricoides* cDNA was subsequently serial diluted tenfold (100, 10, 1 and 0.1 ng), and the standard curve plotted for each dilution point revealed primer efficiency of between 90 and 110%, except for *HSP70* (86.8%) and *INTEGRIN* (138.9%) (Additional file 1: Table S3).

**Table 3:** BLASTX and Blast2GO analyses of putative *Ascaris lumbricoides* virulence-associated genes

Genes	Gene Ontology term analysis (Blast2Go)^a^	Gene description	Species	Identity (%)	E value
*ABA-1*	F: retinol binding	1) ABA-1 allergen 2) ABA-1 allergen 3) Chain A, the solution structure of ABA-1A saturated with oleic acid	*Ascaris lumbricoides* *A. lumbricoides* *A. suum*	100 99 99	6e–144 4e–56 4e–52
*PEBP*	No GO terms	1) Phosphatidylethanolamine-binding-like protein 2) Phosphatidylethanolamine-binding-like protein 3) Phosphatidylethanolamine-binding-like protein	*Toxocara canis* *Ancylostoma duodenale* *Teladorsagia circumcincta*	91 84 78	9e–112 2e–89 7e–88
*CATH-B*	F: cysteine-type endopeptidase activity C: extracellular space C: lysosome P: proteolysis involved in cellular protein catabolic process	1) Cathepsin B-like cysteine proteinase 2) Cathepsin B-like cysteine proteinase 6 3) Papain family cysteine protease	*A. suum* *T. canis* *Haemonchus contortus*	100 84 76	0.0 0.0 0.0
*HSP70*	No GO terms	1) Uncharacterised protein C30C11.4 2) Hypothetical protein WUBG_09411 3) Hypothetical 86.9-kDa protein C30C11.4 in chromosome III, putative	*T. canis* *Wuchereria bancrofti* *Brugia malayi*	91 65 64	0.0 0.0 0.0
*CATH-L*	F: cysteine-type endopeptidase activity F: serine-type endopeptidase activity F: protein binding C: extracellular space C: lysosome C: vesicle lumen C: yolk granule P: proteolysis involved in cellular protein catabolic process	1) Cathepsin L 2) Cathepsin L 3) Cathepsin L family	*A. suum* *T. canis* *Caenorhabditis elegans*	100 89 82	2e–121 4e–107 8e–102
*AQUAPORIN*	F: channel activity C: membrane P: transmembrane transport	1) Aquaporin-3 2) Aquaporin-1 3) Aquaporin-1	*T. canis* *T. canis* *T. canis*	94 93 93	5e–113 0.0 0.0
*VENOM*	C: extracellular region	1) Venom allergen 5 2) *Ancylostoma* secreted protein 3) *Ancylostoma* secreted protein	*T. canis* *T. canis* *T. canis*	58 51 50	6e–30 3e–65 2e–62
*SOD*	F: superoxide dismutase activity F: metal ion binding P: removal of superoxide radicals P: oxidation–reduction process	1) Superoxide dismutase (Mn) 1, mitochondrial 2) Manganese superoxide dismutase 3) Mitochondrial manganese superoxide dismutase	*T. canis* *Ditylenchus destructor* *Bursaphelenchus mucronatus*	90 75 73	5e–131 3e–111 6e–114
*INTEGRIN*	No GO terms	1) Integrin alpha pat-2 2) Integrin alpha pat-2 3) Hypothetical protein WUBG_12493	*T. canis* *Loa loa* *W. bancrofti*	89 78 77	0.0 0.0 1e–155
*CADHERIN*	F: calcium ion binding C: plasma membrane P: homophilic cell adhesion via plasma membrane adhesion molecules C: integral component of membrane	1) Protocadherin-16 2) Cadherin domain protein 3) Cadherin domain containing protein	*T. canis* *Ancylostoma ceylanicum* *H. contortus*	77 33 40	9e–164 3e–11 5e–62
*CLEC*	F: transmembrane signalling receptor activity C: extracellular region C: integral component of plasma membrane F: carbohydrate binding F: monosaccharide binding	1) C-type lectin 2) C-type lectin domain containing protein 3) C-type lectin domain family 4 member E-like	*C. elegans* *H. contortus* *Pelodiscus sinensis*	26 26 33	1e–07 2e–06 1e–06

GO, Gene Ontology

^a^F = molecular function; P = biological process; C = cellular component

**Fig. 3:**
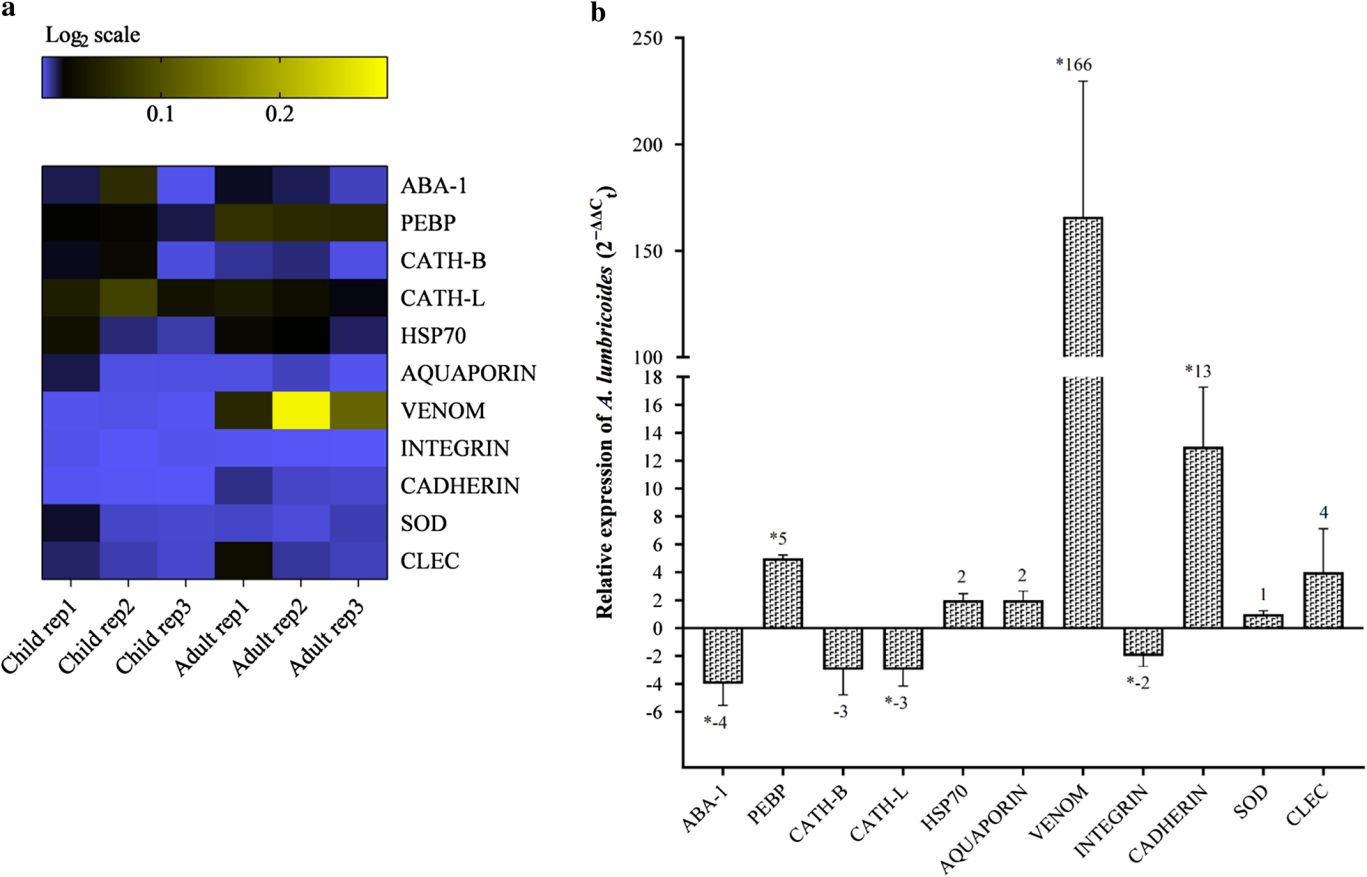
**a** Heat map visualisation of differences in gene expression between putative virulence-associated genes in *A. lumbricoides* from infected adults relative to *A. lumbricoides* from infected children. Coloured scales in the heat map represent log_2_ fold change values, with blue representing downregulated genes and yellow representing upregulated genes. Child rep1, child rep2, child rep3 represent individual *A. lumbricoides* from infected children; adult rep1, adult rep2, adult rep3 represent *A. lumbricoides* from infected adults. **b** Relative expression of putative virulence-associated genes in *A. lumbricoides* from infected adults relative to *A. lumbricoides* from infected children. A normalised ratio (*Y*-axis) representing the 2^−ΔΔCq^ (= 2^−ΔΔCq^) value of > 1 indicates upregulation, whereas a ratio of less than − 1 indicates downregulation. Asterisk indicates a significant difference at *P* < 0.05. The number above the respective bar represents the fold-change for each gene. Note: 2^−ΔΔCq^ represents the difference in ΔC_q_ values between the adult- and children-derived worm samples normalised to reference housekeeping genes

Once the specificity of the newly designed primers was confirmed, qPCR was performed on cDNA prepared from adult worms expelled by the local indigenous adults and children sampled in this study. qPCR data were normalised to the housekeeping or reference genes β-actin [29] and 18S ribosomal RNA [30]. The average C_q_ value for each treatment group was calculated and expression levels were determined using the formula 2^−ΔΔCq^ [31]. As noted earlier, we hypothesised that worms expelled by adults may display higher level of virulence in an attempt to avoid the highly developed adult host defence response. Hence, the expression profile of *A. lumbricoides* from infected adults was compared to that of worms from infected children (Fig. 3a). The comparative analysis demonstrated that *VENOM* was highly upregulated (166-fold, *P* < 0.05) in adults compared to children while the expression of both *CADHERIN* and *PEBP* was elevated significantly (*P* < 0.05) at 13-fold and fivefold, respectively. Conversely, the transcription of *ABA-1* (fourfold), *CATH-L* (threefold) and *INTEGRIN* (twofold) was significantly downregulated (*P* < 0.05) in *A. lumbricoides* from infected adults (Fig. 3b).

## Discussion

To the best of our knowledge, this is the first study to profile the gene expression of *A. lumbricoides* putative virulence-associated genes during infection in adults and children from an endemic area in Malaysia. *Ascaris lumbricoides* is the most common helminthic infection of humans [32]. In highly endemic communities, it is more common to find individuals infected with a small number of worms and only a handful of individuals in the community present with a high worm burden. Parasite establishment in a community is dependent on the dynamics of host–parasite interactions or aggregation whereby only a few individuals harbour high intensity infections [33]. In endemic communities, it is common for re-infection with this parasite to occur within a few months of repeated large-scale administrations of anthelmintic drugs [34]. Information on the pathogenesis of *A. lumbricoides* is limited and mainly implied based on the understanding of its close relative, *A. suum.* Evaluating *A. lumbricoides* virulence by examining the expression of virulence-associated genes during infection may shed light on how this parasite survives in the host as well as provide new targets for drug design and intervention. Hence, the goal of this study was to quantify the expression levels of *A. lumbricoides* putative virulence-associated genes during an infection of adults and children. While many nematode genome sequences are available, information on the *A. lumbricoides* genome at the start of this study was limited to its mitochondrial genome [35]. Given this limitation, the identification of *A. lumbricoides* virulence genes was based on the results from previous studies on *T. canis* to predict *A. suum* homologous sequences. A comparison of the *A. suum* draft genome sequence [21] with that of other species indicated that *T. canis* genes had the highest sequence similarity to *A. suum* [26]. With the recent publication of the *A. lumbricoides* genome (Acc. No. SMSY010000002.1) [36], we were able to confirm that the sequences of the selected *A. lumbricoides* genes are between 96.4 and 100% identical to the *A. suum* gene sequences.

Over time, parasites have developed a remarkable set of molecular adaptations that manipulate, inhibit or activate different host cells or pathways to maximise their success in the host [12, 15]. Numerous groups of proteins have been suggested to play major biological roles in host–parasite interactions [37], including the sperm-coat protein (SCP)-like extracellular proteins (SCP/TAPS proteins). This family of proteins is also called SCP/Tpx-1/Ag5/PR-1/Sc7 (SCP/TAPS; Pfam: PF00188) and belongs to the cysteine-rich secretory protein (CRISP) superfamily [38, 39]. In the present study, expression of the *A. lumbricoides* venom allergen 3 (*VENOM*) gene was significantly upregulated, at 166-fold (*P* < 0.05), in worms isolated from infected adults compared to *A. lumbricoides* isolated from infected children. The venom allergen protein is homologous to the SCP/TAPS protein [16, 40]. SCP/TAPS homologues have been characterised for a range of helminth species, including *Schistosoma mansoni* [38], *Ancylostoma caninum* [19, 41], *Necator americanus* [17], *Haemonchus contortus* [42], *Onchocerca volvulus* [43, 44], *T. canis* [26, 45], and *Brugia malayi* [16].

In *A. caninum*, SCP/TAPS proteins are known as activation-associated proteins (ASPs). There are two types of ASPs: double- and single-domain ASPs, designated as Ac-ASP-1 and Ac-ASP-2, respectively. These proteins are secreted in response to host-specific signals during the infection process [46]. More recently, four additional ASPs have been characterised in adult *A. caninum*, namely Ac-ASP-3, Ac-ASP-4, Ac-ASP-5 and Ac-ASP-6, while Ac-ASP-7 is highly expressed in the *A. caninum* larvae during transition from the free-living to parasitic stage [47]. In addition, another study on *A. caninum* ASPs revealed that these proteins are the most abundantly characterised in serum-activated third-stage larvae (L3s) [48] and ASPs also dominate the ES proteins released by these worms [49]. Another SCP/TAPS molecule, referred to as neutrophil inhibitory factor (NIF), was first isolated from adult *A. caninum* and has been shown to play an immunomodulatory role by blocking the adhesion of activated neutrophils to vascular endothelial cells and the subsequent release of H_2_O_2_ from activated neutrophils [50]. In addition, Ac-ASP-1 and Ac-ASP-2 homologues have also been identified in the L3s of *N. americanus* (Na-ASP-1 and Na-ASP-2) [17, 51]. The Na-ASP-2 protein has structural and charge similarities to CC-chemokines, suggesting that it might act as a chemokine mimic when released by the infective larvae during tissue migration [52]. For *B. malayi*, the only SCP/TAPS homologue that has been characterised to date is Bm-VAL-1. Nonetheless, the expression of Bm-VAL-1 was reported to be restricted to L3s, which raises the question of its role in host invasion [16]. SCP/TAPS molecules identified in *Caenorhabditis elegans* [19] have been shown to be involved mainly in biological processes, such as antimicrobial activity [53], regulation of longevity, stress resistance [54] and normal fat storage [55]. Thus, the high-level expression of *VENOM* in *A. lumbricoides* from infected adults suggests that this protein plays a significant role during infection. However, whether the protein is expressed at the point of initiation, establishment or maintenance during infection warrants further study.

The second highly expressed putative virulence-associated gene in *A. lumbricoides* from infected adults was *CADHERIN*, expressed 13-fold higher (*P* < 0.05) than in worms from infected children. Cadherins form a superfamily of transmembrane glycoproteins involved in calcium-dependent cell–cell adhesion. These molecules are one of five classes of cell adhesion molecules (CAMs) [56]. The cadherin superfamily includes classical cadherins, protocadherins and atypical cadherins [57]. Generally, cadherins play a significant role in tissue morphogenesis and homeostasis [58, 59], although information on cadherins in parasitic nematodes is limited. Cadherins have been identified in *C. elegans*, with 12 genes encoding 13 cadherins [60, 61]. The main cadherin families that are conserved throughout metazoans include members of the classical cadherin, fat-like cadherin, dachsous, flamingo/CELSR and calystenin families [62]. Unlike other invertebrates, *C. elegans* lacks protocadherin [63]. The classical cadherin–catenin complex is essential for diverse morphogenetic events during embryogenesis through its interactions with a range of mostly conserved proteins that act to modulate its function. While the other members of the cadherin family in *C. elegans* are not well characterised, they play clear roles in neuronal development and function [62]. Meanwhile, analysis of the *T. canis* genome predicted that the *T. canis* secretome contains at least 870 ES proteins that are proposed to play different roles in host–parasite interaction. Among the ES proteins, 23 are CAMs, including cadherin [26]. In addition, recent comparative genomics analysis of *Trichinella spiralis* has shown that the cadherin families may be involved in structural remodelling during nurse cell formation [64]. While these molecules are associated with biological processes in *C. elegans* and most metazoans, the upregulation of *CADHERIN* in this study does not necessarily imply that it is a virulence factor of *A. lumbricoides*, as the underlying mechanism of this molecule is poorly understood, particularly in parasitic helminths. Hence, within this context, the expression of *CADHERIN* may be associated with cell–cell adhesion but further characterisation is required.

The gene encoding for phosphatidylethanolamine-binding protein (*PEBP*) was also significantly upregulated (fivefold) in *A. lumbricoides* during the infection of adult individuals. *PEBP* is highly conserved in organisms, including bacteria, yeast, plants, nematodes, *Drosophila* and mammals [65]. *PEBP* controls several signalling pathways, such as inhibition of the MAPK pathway [65] and the NF-κB pathway [66], regulation of heterotrimeric G proteins [67] and serine protease inhibition [68]. In *T. canis*, *PEBP*, formerly known as TES-26 but later renamed Tc-PEB-1 [25], is part of the ES proteins and predicted to be involved in immune evasion [18, 26]. Meanwhile, in *Trichuris muris* (mouse whipworm), Tm16, a whipworm ES protein, has been identified and, based on its amino acid sequence, assigned to the PEBP superfamily. The structure of Tm16 revealed a prototypical phosphatidylethanolamine-binding-like topology with a large binding cavity capable of accommodating various ligands. This is suggestive of its ability to bind to macromolecules related to the signalling pathway and to transduction or cell migration and regulation. Moreover, it also shares a similar structure to human PEBP (hPEBP) and may have similar functions [69]. Based on evidence from the *T. muris* study, we suggest that *A. lumbricoides* *PEBP* also plays a role in the survival of the parasite in the host.

It is possible that the statistically significant expression of *VENOM*, *CADHERIN* and *PEBP* seen in *A. lumbricoides* from infected adults relative to *A. lumbricoides* from infected children could be a result of differences in the host–parasite interaction between adults and children. The immune system is well developed in adults and works efficiently to maintain our defences against many types of pathogens. Similar to other helminth infections, *A. lumbricoides* induces a highly polarised Th2 immune response [70]. Chronic infections with *A. lumbricoides* in humans are associated with the production of high levels of specific and nonspecific antibodies of all isotypes and immunoglobin (Ig) G subclasses [71, 72], and cytokine response is characterised by the production of Th2 cytokines such as interleukin-4 (IL-4), IL-13, and IL-15 by peripheral blood monocytes and leukocytes [70, 73, 74]. Additionally, a specific IgE has a role in protective immunity in *A. lumbricoides* infection, either in the initiation of allergic-type inflammatory responses against the parasite or in the amplification of other Th2-mediated mechanisms [75]. However, several other studies have established a clear negative association between parasite-specific IgE levels and infection intensity [76].

The intensity of the infection may depend on various factors, such as host genetics and/or the infected individual’s socio-economic or nutritional status and also the host acquired immune response through previous exposure to the parasite. The innate and adaptive immune systems of young children mature more gradually, making children more vulnerable to many pathogens, including parasitic helminths. Nonetheless, adults are also capable of harbouring *A. lumbricoides*, although with fewer worms than children. This implies a slow build-up of specific immunity or variation in susceptibility to infection over time [77]. In this study, expelled worms were obtained from children aged ≤ 5 years. The intestinal phase of infection is usually asymptomatic, although moderately heavy infections can affect the health, growth and physical fitness of children [78]. It is suggested that children display more symptoms due to the infection compared to adults as their immune system and memory are still underdeveloped to fight the infection, which explains the lower expression of the putative virulence-associated genes observed in this study. A parallel study on the expression of host immune-related genes during *A. lumbricoides* infection would provide a better understanding of host–parasite interactions.

As individuals get older, they develop an expanding repertoire of memory T and B cells triggered by previous infections [23]. As such, we propose that the immune system of the adult hosts participating in the present study has been primarily moulded by evolution as a result of repeated infection by *A. lumbricoides*, allowing the adults to fight off the infection. However, the helminths are well adapted to their definitive host and adopt various strategies, such as modulating the expression of virulence genes, to overcome or restrict the host defence system by manipulating and modulating host immunity, leading to a failure of the host to eliminate the parasite [14]. One of these virulence factors may be *VENOM*, which was significantly upregulated in the adult indigenous individuals sampled. As noted above, *VENOM* is homologous to the SCP/TAPS protein in other parasitic worms and SCP/TAPS proteins are activated in response to host-specific signals produced during the infection process [46].

## Conclusion

In conclusion, we have shown that the selected *A. lumbricoides* putative virulence-associated genes are overexpressed during an active infection of humans and that expression levels are higher in worms expelled by adults compared to children. Whether the proteins encoded by the significantly overexpressed putative genes (i.e. *VENOM*, *CADHERIN* and *PEBP*) play key biological roles in the infected human host remains to be confirmed. Further studies on host cell mechanisms manipulated by these proteins could facilitate the discovery of new drugs and identification of vaccine targets.

## Supplementary Information

**Additional file 1: Table S1. **Prevalence of STH infections according to the intensity of infections based on Kato-Katz protocol. **Table S2. **Predicted function(s) of putative *A. lumbricoides* virulence-associated genes. **Table S3. **Primer efficiency for each of the 11 *A. lumbricoides *putative virulence-associated genes.

## Data Availability

The datasets supporting the conclusions of this article are included within the article and its Additional files.

## Abbreviations

C_q_: Quantification cycle
ES: Excretory/secretory
GO: Gene ontology
PEBP: Phosphatidylethanolamine-binding protein
SCP/TAPS: Sperm-coat proteins/Tpx-1/Ag5/PR-1/Sc7
STH: Soil-transmitted helminth
