# Supplementary material for: Expression of Ascaris lumbricoides putative virulence-associated genes when infecting a human host
Source: Parasit Vectors. 2021 Mar 23;14:176. doi: 10.1186/s13071-021-04680-y (PMC7985925; doi:10.1186/s13071-021-04680-y)
Supplement: Supplementary file 1 — Additional file 1: Table S1. Prevalence of STH infections according to the intensity of infections based on Kato-Katz protocol. Table S2. Predicted function(s) of putative A. lumbricoides virulence-associated genes. Table S3. Primer efficiency for each of the 11 A. lumbricoides putative virulence-associated genes. [file 13071_2021_4680_MOESM1_ESM.docx]

**SUPPLEMENTARY INFORMATION**

| **Intensity of infections** | **Type of infections** | | | | | | | | |
| --- | --- | --- | --- | --- | --- | --- | --- | --- | --- |
|  | ***Trichuris trichiura*** | | | ***Ascaris lumbricoides*** | | | **Hookworm** | | |
|  | **No.** | **%** | **Median**  **(IQR)** | **No.** | **%** | **Median**  **(IQR)** | **No.** | **%** | **Median**  **(IQR)** |
| Light infections | 37 | 9.0 | 266  (22, 644) | 21 | 5.1 | 44  (22, 3,774) | 4 | 1 | 44  (22, 44) |
| Moderate infections | 45 | 10.9 | 2,042  (1,088, 8,791) | 9 | 2.2 | 16,250  (5,417, 40,892) | 1 | 0.2 | 155  (155, 155) |
| Heavy infections | 0 | 0.0 | N/A | 5 | 1.2 | 122,677  (65,490, 24,5310) | 0 | 0 | N/A |
| Total examined | 82 | 20.0 |  | 35 | 8.5 |  | 5 | 1.2 |  |
| Missing * | 329 | 80.0 |  | 376 | 91.5 |  | 406 | 98.8 |  |
| Median (IQR) | 1,188 (283, 2,675) | | | 2331 (44, 26,152) | | | 44 (33, 100) | | |

**Table S1**

Prevalence of STH infections according to the intensity of infections based on Kato-Katz protocol

*N/A: not available

IQR: interquartile range

**Table S2**

Predicted function(s) of putative *A. lumbricoides* virulence-associated genes**.**

| Genes | Immunological effects | Species |
| --- | --- | --- |
| ABA-1 allergen | 1. Parasite allergen that belongs to a family of fatty acid binding proteins and binds to different lipids and retinoids (Kennedy et al., 2011; Xia et al., 2000) | *A. lumbricoides* |
| Phosphatidylethanolamine-Binding Proteins | 1. Known as Tm16, may have the ability to bind to macromolecules related to signalling pathways and transduction or cell migration and regulation (Liu et al., 2017) 2. Known as TES-26 (Tc-PEB-1), part of the excretory/secretory (ES) proteins and predicted to be involved in immune evasion (Maizels et al., 2000; Zhu et al., 2015) | *T. muris*  *T. canis* |
| Cathepsin-B | 1. May be involved in invasion of the central nervous system during host-parasite interaction and penetration into the host gut wall (Han et al., 2011; Long et al., 2015) 2. Cathepsin B-like involved in digestion (Jasmer et al., 2015) 3. Cathepsin-B1 involved in tissue degradation (Williamson et al., 2003) | *Angiostrongylus cantonensis*  *A. suum*  *A. caninum* |
| Cathepsin-L | 1. Displays a differential ability to degrade host collagen (Robinson et al., 2011) 2. Degradation of tissues and IgG (Li et al., 2006) | *F. hepatica*  *Taenia solium* |
| Heat Shock Protein 70kDa | 1. Displays strong thermo tolerance and might be an important molecule in parasite-host interaction (Yang et al., 2012) 2. The expression of HSP70 can be fleetingly induced by stress and temperature during parasite transformation (Neumann et al., 1993) | *Schistosoma japonicum*  *S. mansoni* |
| Aquaporin 3 | 1. Known as TcAQP1 and might play a role in the drug uptake. The predominant expression of TcAQP1 in the intestine of adult *T. canis* indicates an important of this protein in parasite survival (Ma et al., 2019). 2. TcAQP1 predicted to enable transport of water and solutes (Luo et al., 2016) | *T. canis*  *T. canis* |
| Venom allergen 3 | 1. Known as ASPs secreted in response to host-specific signals during the infection process (Bethony et al., 2005) 2. Known as NIF, blocks adhesion of activated neutrophils to vascular endothelial cells and the subsequent release of H_2_O_2_ from activated neutrophils (Moyle et al., 1994) 3. Known as Na-ASP-2, might act as a chemokine mimic when released by the infective larvae during tissue migration (Bower et al., 2008) | *A. caninum*  *A. caninum*  *N. americanus* |
| Integrin alpha | 1. Predicted cell-adhesion molecule (Zhu et al., 2015) | *T. canis* |
| Cadherin | 1. Predicted cell-adhesion molecule (Zhu et al., 2015) 2. May be involved in structural remodelling during the formation of nurse cells (Qu et al., 2019) | *T. canis*  *T. spiralis* |
| Superoxide dismutase | 1. Possible key pro-active defence factor against reactive oxygen species (ROS) produced by effector cells as part of the host immune response (Taiwo et al., 1999) | *N. americanus* |
| C-type lectin | 1. Known as TES-70, is able to bind mammalian carbohydrates in a calcium-dependent manner – inhibits the migration of host cells; masks worm carbohydrates from host immune cells (Loukas et al., 2000) 2. Known as As-CTL-1, this protein may facilitate avoidance of host pathogen recognition mechanisms due to its similarity to host immune cells receptors (Yoshida et al., 2012) | *T. canis*  *A. suum* |

**Table S3**

Primer efficiency for each of the 11 *A. lumbricoides* putative virulence-associated genes

| **Gene** | **Primer Efficiency (%)** |
| --- | --- |
| ABA-1 | 94.9 |
| PEBP | 90.9 |
| CATH-B | 105.7 |
| CATH-L | 92.2 |
| HSP70 | 86.8 |
| AQUAPORIN | 102 |
| VENOM | 96.2 |
| INTEGRIN | 136.9 |
| CADHERIN | 107.2 |
| SOD | 95.2 |
| CLEC | 91.1 |

**References**

Bethony, J., Loukas, A., Smout, M., Brooker, S., Mendez, S., Plieskatt, J., Goud, G., Bottazzi, M.E., Zhan, B., Wang, Y., Williamson, A., 2005. Antibodies against a secreted protein from hookworm larvae reduce the intensity of hookworm infection in humans and vaccinated laboratory animals. FASEB J. 19 (12), 1743–1745.

Bower, M.A., Constant, S.L., Mendez, S., 2008. *Necator americanus*: the Na-ASP-2 protein secreted by the infective larvae induces neutrophil recruitment in vivo and in vitro. Exp. Parasitol. 118 (4), 569–575.

Han, Y.P., Li, Z.Y., Li, B.C., Sun, X., Zhu, C.C., Ling, X.T., Zheng, H.Q., Wu, Z.D., Lv, Z.Y., 2011. Molecular cloning and characterization of a cathepsin B from *Angiostrongylus cantonensis*. Parasitol. Res. 109 (2), 369–378.

Jasmer, D.P., Rosa, B.A., Mitreva, M., 2015. Peptidases compartmentalized to the *Ascaris suum* intestinal lumen and apical intestinal membrane. PLoS Negl. Trop. Dis. 9 (1), e3375.

Kennedy, M.W., 2011. The polyprotein allergens of nematodes (NPAs)–Structure at last, but still mysterious. Exp. Parasitol. 129 (2), 81–84.

Li, A.H., Moon, S.U., Park, Y.K., Na, B.K., Hwang, M.G., Oh, C.M., Cho, S.H., Kong, Y., Kim, T.S., Chung, P.R., 2006. Identification and characterization of a cathepsin L-like cysteine protease from *Taenia solium* metacestode. Vet. Parasitol. 141 (3–4), 251–259.

Liu, Z., Kelleher, A., Tabb, S., Wei, J., Pollet, J., Hotez, P.J., Bottazzi, M.E., Zhan, B., Asojo, O.A., 2017. Identification, characterization, and structure of Tm16 from *Trichuris muris*. J. Parasitol. Res. 2017, 4342789.

Long, Y., Cao, B., Yu, L., Tukayo, M., Feng, C., Wang, Y., Luo, D., 2015. *Angiostrongylus cantonensis* cathepsin B-like protease (Ac-cathB-1) is involved in host gut penetration. Parasite, 22.

Loukas, A., Doedens, A., Hintz, M., Maizels, R.M., 2000. Identification of a new C-type lectin, TES-70, secreted by infective larvae of *Toxocara canis*, which binds to host ligands. Parasitology 121 (5), 545–554.

Luo, Y.F., Hu, L., Ma, G.X., Luo, Y.L., Yin, S.S., Xiong, Y., Zhu, X.Q., Zhou, R.Q., 2016. Characterization and differential expression analysis of *Toxocara canis* aquaporin-1 gene. Parasitol. Res. 115 (9), 3631–3636.

Ma, G., Jiang, A., Luo, Y., Luo, Y., Huang, H., Zhou, R., 2019. Aquaporin 1 is located on the intestinal basolateral membrane in *Toxocara canis* and might play a role in drug uptake. Parasites Vectors 12 (1), 243.

Maizels, R.M., Tetteh, K.K., Loukas, A., 2000. *Toxocara canis*: genes expressed by the arrested infective larval stage of a parasitic nematode*.*Int. J. Parasitol. 30 (4), 495–508.

Moyle, M., Foster, D.L., McGrath, D.E., Brown, S.M., Laroche, Y., De Meutter, J., Stanssens, P., Bogowitz, C.A., Fried, V.A., Ely, J.A., 1994. A hookworm glycoprotein that inhibits neutrophil function is a ligand of the integrin CD11b/CD18. J. Biol. Chem. 269 (13), 10008–10015.

Neumann, S., Ziv, E., Lantner, F., Schechter, I., 1993. Regulation of HSP70 gene expression during the life cycle of the parasitic helminth *Schistosoma mansoni*. Eur. J. Biochem. 212 (2), 589–596.

Qu, Z., Li, W., Zhang, N., Li, L., Yan, H., Li, T., Cui, J., Yang, Y., Jia, W., Fu, B., 2019. Comparative genomic analysis of *Trichinella spiralis* reveals potential mechanisms of adaptive evolution. Biomed Res. Int. 2019.

Robinson, M.W., Corvo, I., Jones, P.M., George, A.M., Padula, M.P., To, J., Cancela, M., Rinaldi, G., Tort, J.F., Roche, L., Dalton, J.P., 2011. Collagenolytic activities of the major secreted cathepsin L peptidases involved in the virulence of the helminth pathogen, *Fasciola hepatica.* PLoS Negl, Trop, Dis. 5 (4), e1012.

Taiwo, F.A., Brophy, P.M., Pritchard, D.I., Brown, A., Wardlaw, A., Patterson, L.H., 1999. Cu/Zn superoxide dismutase in excretory–secretory products of the human hookworm *Necator americanus*: An electron paramagnetic spectrometry study. Eur. J. Biochem. 264 (2), 434–438.

Williamson, A.L., Brindley, P.J., Knox, D.P., Hotez, P.J., Loukas, A., 2003. Digestive proteases of blood-feeding nematodes. Trends Parasitol. 19 (9), 417–423.

Xia, Y., Spence, H.J., Moore, J., Heaney, N., McDermott, L., Cooper, A., Watson, D.G., Mei, B., Komuniecki, R., Kennedy, M.W., 2000. The ABA-1 allergen of *Ascaris lumbricoides*: sequence polymorphism, stage and tissue-specific expression, lipid binding function, and protein biophysical properties. Parasitology 120 (2), 211–224.

Yang, J., Yang, L., Lv, Z., Wang, J., Zhang, Q., Zheng, H., Wu, Z., 2012. Molecular cloning and characterization of a HSP70 gene from *Schistosoma japonicum*. Parasitol. Res. 110 (5), 1785–1793.

Yoshida, A., Nagayasu, E., Horii, Y., Maruyama, H., 2012. A novel C-type lectin identified by EST analysis in tissue migratory larvae of *Ascaris suum*. Parasitol. Res. 110 (4), 1583–1586.

Zhu, X.Q., Korhonen, P.K., Cai, H., Young, N.D., Nejsum, P., von Samson-Himmelstjerna, G., Boag, P.R., Tan, P., Li, Q., Min, J., Yang, Y., 2015. Genetic blueprint of the zoonotic pathogen *Toxocara canis*. Nat. Commun. 6 (1), 1–8.
